# Sleep duration and sleep disturbances in association with falls among the middle-aged and older adults in China: a population-based nationwide study

**DOI:** 10.1186/s12877-018-0889-x

**Published:** 2018-08-28

**Authors:** Samuel Kwaku Essien, Cindy Xin Feng, Wenjie Sun, Marwa Farag, Longhai Li, Yongqing Gao

**Affiliations:** 1 School of Public Health, University of Saskatchewan, Health Sciences Building E-Wing, 104 Clinic Place, Saskatoon, SK S7N 2Z4 Canada; 2 Robert Stempel College of Public and Social Work, Florida international University, Miami, FL 33199 USA; 3 School of Food Science, Guangdong Pharmaceutical University, Zhongshan, 528458 China; 4 Department of Mathematics and Statistics, University of Saskatchewan, Saskatoon, SK S7N 5E6 Canada

**Keywords:** Falls, Sleep duration, Sleep disturbance, Middle-aged and older adults

## Abstract

**Background:**

Falls pose major health problems to the middle-aged and older adults and may potentially lead to various levels of injuries. Sleep duration and disturbances have been shown to be associated with falls in literature; however, studies of the joint and distinct effects of those sleep problems are still sparse. To fill this gap, we aimed to determine the association between sleep duration, sleep disturbances and falls among middle-aged and older adults in China controlling for psychosocial, lifestyle, socio-demographical factors and comorbidity.

**Methods:**

Data were derived from the China Health and Retirement Longitudinal Study (CHARLS) based on multi-stage sampling designs, with respondents aged 50 and older. Associations were evaluated by using multiple logistic regression adjusting for confounders and complex survey design. To further determine if the association of sleep duration/disturbance and falls depends on age groups, the study data were divided into two samples (age 50–64 vs. age 65+) and comparison was made between the two age groups.

**Results:**

Of the 12,759 respondents, 2172 (17%) had falls within the last 2 years. Our findings indicated that the participants who had nighttime sleep duration ≤5 were more likely to report falls than those who had nighttime sleep duration ≥6 h; whereas no association between nighttime sleep duration > 8 h and falls. Participants having sleep disturbances 1–2 days, or 3–4 days, and 5–7 days per week were also more likely to report falls than those who had no sleep disturbance. The nap sleep duration was not significantly associated with falls. Although the combined sample found both sleep duration and sleep disturbance to be strongly associated with falls after adjusting for various confounders**,** sleep disturbance was not significantly related to falls among participants aged 65 + .

**Conclusions:**

Our study suggested that there is an independent association between falls and short sleep duration and disturbed sleep among middle-aged and older adults in China. Findings underscore the need for evidence-based prevention and interventions targeting sleep duration and disturbance among this study population.

## Background

Falls pose major health problems to the middle-aged and older adults and may potentially lead to various levels of injuries, disability [1], death [2], and hospitalizations [3]. Increased prevalence of falls in elderly populations per year has been reported globally, such as 31% in the United States [4], 20–30% in Canada [5], 25.6% in Australia [6] and 27.6% in Brazil [7]. In China, the aging population has increased rapidly over the past two decades [8], contributing to China having the “largest aging population in the world” [9]. Out of over 160 million Chinese aged 60 and above globally, 99% currently reside in China [10]. The annual prevalence of falls among the Chinese elderly has been reported to range from 6 to 31% according to WHO global report on fall [11]. Increased fall rate among middle-aged and older adults comes with high economic repercussions in terms of direct and indirect cost. It was reported that the economic cost associated with fall-related health problems is about 8.7 billion CAD dollars in Canada [12], 19.2 billion US dollars in the United States [13] and 500 million dollars in Australia [14]. In China, an estimated yearly cost of falls in elderly Chinese is projected to be between 1.6–1.8 billion Chinese Yuan Renminbi, which is 1.5 times higher than the projected cost for the adolescent-adult [14].

Despite the evidence, both intrinsic (e.g., depression, impaired vision, gender) and extrinsic (e.g., medication especially polypharmacy, poor lighting, footwear) factors are associated with falls [15], limited research has been conducted focusing on the Chinese middle-aged and older adults. The elderly Chinese represent the population at greatest risk of falls in China [14], who are more vulnerable to the risk factors attributable to falls, such as poor sleep [16, 17], feeling lonely because of “their greater rates of widowhood” [18] and other lifestyles including alcohol consumption and smoking [19]. It has been reported that large proportion of Chinses older persons are prone to sleep inadequacies and poor sleep quality [20, 21]. For instance, Liu et found that almost 53% of Chinese older persons experience “more than one day per week” poor quality sleep [20]. This highlights the vulnerability to poor sleep quality experienced by this group. As such, identifying and understanding the risk factors associated with falls in the Chinese middle-aged and older adults is imperative for designing effective prevention and intervention strategies for falls.

One primary risk factor that has been shown to be associated with falls is sleep duration; however, the evidence in literature is mixed with some agreeing an association between shorter sleep duration (≤ 5 h) and falls [22–25] exists and others suggesting an association between longer sleep duration (> 8 h) and falls [23, 25, 26]. Ascertaining the importance of good sleep (e.g. sleep duration/quantity) especially in larger samples with varied age range and gender, has been encouraged in sleep-related research [27]. Sleep quality might also be characterized as sleep disturbance, which has been shown to be associated with the risk of falling [28, 29]. Sleep disturbance may be attributable to environment factors such as exposure to noise [30, 31]. Other non-environmental factors such as myocardial infarction [32, 33] and obstructive sleep apnea [34] have also been reported to be associated with sleep disturbance/poor sleep. However, these factors were beyond the scope of the data source used for the current study analyses. Investigating sleep duration effect independently in middle-aged and older adults may be ideal, but since the association between sleep duration and sleep disturbance is unclear [35], accounting for both simultaneously is necessary as they may have some distinct features impacting on the risk of falls.

Better understanding of the relationship between sleep duration and disturbance with falls will shed light on designing the evidence-based prevention and interventions targeting this vulnerable population. Therefore, the objective of this study is to investigate association of sleep duration and disturbance with falls using a national-wide representative data focusing on the middle-aged and older adults in China, while adjusting for various psychosocial, lifestyle, socio-demographical factors and comorbidity.

## Methods

The national baseline data from the China Health and Retirement Longitudinal Study (CHARLS) was used to investigate the association between sleep duration, sleep disturbances and falls in Chinese middle-aged and older adults adjusting for psychosocial, lifestyle, socio-demographical factors and comorbidity. The CHARLS baseline data collection took place in June 2011 and March 2012 on 17,708 respondents in 10,257 households [36, 37] and the target population was middle-aged and older adults and their spouse aged 45 years and above and currently residing in a household in China [9, 36]. The CHARLS national survey commenced after the Health and Retirement Study have been introduced and conducted in the United States [9, 37]. The survey was pilot tested on a sample of middle-aged and older adults and their spouse from 95 communities in 32 counties [9] to test for face validity. A complex survey structure consisting of a four-stage, stratified, cluster probability sampling design was used to collect the CHARLS data [9, 38]. The data contains information on the socio-demographics, family, health status, health care, employment, and the household economy. Health-related questions included self-reported health status, previous medical history, lifestyle, health behaviors, and activities of daily living. However, for the purpose of the present study, all analyses focused on 13,727 of participants aged 50 and over. Ethical approval for collecting data on human subjects was received at Peking University by their institutional review board (IRB). The CHARLS data are publicly available from the China Health and Retirement Longitudinal Study website:  http://charls.pku.edu.cn/en.

### Fall

The primary outcome of interest in this study is fall. Participants were asked to respond “yes” or “no” to the question “Have you fallen down in the last two years?”

### Sleep duration and sleep disturbance

Sleep duration was assessed with the question “During the past month, how many hours of actual sleep did you get at night?” which was recorded as an integer. The sleep duration question was adapted from the Pittsburgh Sleep Quality Index (PSQI) [20, 39, 40]. The reliability and validity of the questions in the Pittsburgh Sleep Quality Index (PSQI) has been reported elsewhere [39, 41]. Following the literature [22–25], sleep duration was categorized in “≤5 h”, “6–7 h”, “8 h”, “9–10 h” and “≥11” at night in the past month. Sleep disturbance from combined sources of having trouble falling asleep [26], frequently nighttime awakenings and earlier waking [42] was measured in days per week on a scale of four: “none (<1 day)”, “little (1-2 days)”, “occasionally (3-4 days)” and “most (5-7 days)”. Besides sleep duration at night and sleep disturbance, participants were also asked to report time in minutes to the question “During the past month, how long did you take a nap after lunch?”

### Psychosocial, lifestyle, sociodemographic factors and comorbidity

Psychological wellbeing was assessed with the self-report question “how you have felt during the last week” for depression and anxiety (fear) with the following options: “none (<1 day per week)”, “little (1-2 days per week)”, “occasionally (3-4 days per week)” and “most (5-7 days per week)”. Marital status was reported as married with spouse present, married not living with spouse, separated divorced and widowed and never married. Participants were classified into individuals who had never exposed to alcohol and participants who had exposed to alcohol at some time in their lives. Frequent smoking was rated as never smoker, past smoker and current smoker. Age of participants were categorized into three levels: “50–64 years”, “65–74”, and “75 years and over”. Body mass index (BMI) was computed as current weight in kilograms according to self-reporting in postal survey divided by the square of height in meters. In addition, fall related comorbidities available in the data including diabetes, stroke [43], visual impairment/problem [44], mental impairment [45] and arthritis [46] were considered. Diabetes, hypertension, stroke and arthritis were accessed as “yes/no” based on the question “Have you been diagnosed with conditions listed below by a doctor?” Visual and mental impairments were also accessed on the question “Do you have one of the following disabilities?” and participants answered “yes/no” to the question.

To further determine if the association of sleep duration/disturbance and falls depends on age groups, the study data were divided into two samples (age 50–64 vs. age 65+) and comparison was made between the two age groups.

### Data analysis

The variables were screened by examining the unconditional association between each risk factor and the outcome using logistic regression. Variables where the *p*-value < 0.25 based on the type 3 Wald test were retained for consideration in building the final model [47]. The Akaike information criterion (AIC) [48] was then used to decide whether to include a variable as continuous or categorical. Manual backward selection was used to develop a main effects model, retaining only variables where *p*-value < 0.05. A variable was considered a confounder “if the adjusted estimate is different from the crude estimate” by at least 20% [49]. All possible interactions were tested among all predictors that are significant in the final main effects model. All the analyses were performed using *proc surveylogistic* in SAS 9.4 to properly account for the complex survey sampling structures, including designs, stratification, clustering, and unequal weighting [50]. The associations between covariates and falls in the final model were reported as adjusted odds ratios (AOR) with 95% confidence intervals (95% CI) and *p*-values.

## Results

Of the 12,759 middle-aged and older adults who responded questions on falls, 2172 (17%) had falls within the last 2 years.

The characteristics of the current study sample (see Table 1) revealed almost 32% of the participants had sleep duration no more than 5 h per day, less than half of the participants, about 40% had sleep duration between 6 and 7 h per day and almost 21% had sleep duration 8 h per day. Among those who had sleep duration no more than 5 h, about 22% had falls, whereas only 13% participants had falls for those who had sleep duration of 8 h. For sleep disturbance, almost 20% of the participants had sleep disturbance 5–7 days per week, 15.4% had 3–4 days per week, 16.7% had 1–2 days per week and 47.6% reported less than 1-day sleep disturbance. Among those who had sleep disturbance 5–7 days per week, almost 23% had falls as compared to 13.5% for those who reported less than 1-day sleep disturbance.

**Table 1:** Descriptive statistics and *p*-values computed from the bivariate analysis

Variables	Total (%)	Fall		*p-*value
		Yes (%)	No (%)	
*Sleep duration* ^*a*^ *(in hours) (n = 12,629)*				
≤ 5	3971 (31.4)	878 (22.1)	3093 (77.9)	< 0.0001
6–7	5008 (39.7)	773 (15.4)	4235 (84.6)	
8 (ref)	2602 (20.6)	338 (13.0)	2264 (87.0)	
9–10	943(7.5)	138 (14.6)	805 (85.4)	
≥ 11	105 (0.8)	22 (20.9)	83 (79.1)	
*Sleep disturbance* ^*b*^ *(n = 12,562)*				
None (> 1 day) (ref)	5983 (47.6)	810 (13.5)	5173 (86.5)	< 0.0001
Little (1–2 days)	2098 (16.7)	354 (16.9)	1744 (83.1)	
Occasionally (3–4 days)	1932 (15.4)	377 (19.5)	1555 (80.5)	
Most (5–7 days)	2549 (20.3)	595 (23.3)	1954 (76.7)	
*Age in years (n = 12,739* ***)***				
50–64 (ref)	8331 (65.4)	1271 (15.3)	7060 (84.7)	< 0.0001
65–74	3045 (23.9)	598 (19.6)	2447 (80.4)	
75+	1363 (10.7)	299 (21.9)	1064 (78.1)	
*Gender (n = 12,751)*				
Female (ref)	6533(51.2)	1265(19.4)	5268 (80.6)	< 0.0001
Male	6218 (48.8)	907 (14.6)	5311 (85.4)	
*Smoking (n = 12,745)*				
Never (ref)	7554 (59.3)	1343 (17.8)	6211 (82.2)	0.0049
Past	1241 (9.7)	218 (17.6)	1023 (82.4)	
Current	3950 (31.0)	610 (15.4)	3340 (84.6)	
*Drinking Behaviour (n = 12,747)*				
No intake of alcohol	4081 (32.0)	722 (17.7)	3359 (82.3)	0.2380
Exposed to alcohol intake	8666 (68.0)	1449 (16.7)	7217 (83.3)	
*Depression* ^*b*^ *(n = 12,450)*				
None (< 1 day) (ref)	5630 (45.2)	745 (13.2)	4885 (86.8)	< 0.0001
Little (1–2 days)	2830 (22.7)	449 (15.9)	2381 (84.1)	
Occasionally (3–4 days)	2361 (19.0)	483 (20.5)	1878 (79.5)	
Most (5–7 days)	1629 (13.1)	432 (26.5)	1197 (73.5)	
*Anxiety* ^*b*^ *(n = 12,544)*				
None (> 1 day) (ref)	9905 (78.9)	1569 (15.8)	8336 (84.2)	< 0.0001
Little (1–2 days)	1299 (10.4)	238 (18.3)	1061 (81.7)	
Occasionally (3–4 days)	786 (6.3)	172 (21.9)	614 (78.1)	
Most (5–7 days)	554 (4.4)	151 (27.3)	403 (72.7)	
*Loneliness* ^*b*^ *(n = 12,497)*				
None (> 1 day) (ref)	8650 (69.2)	1311 (15.2)	7339 (84.8)	< 0.0001
Little (1–2 days)	1598 (12.8)	287 (18.0)	1311 (82.0)	
Occasionally (3–4 days)	1189 (9.5)	258 (21.7)	931 (78.3)	
Most (5–7 days)	1060 (8.5)	264 (24.9)	796 (75.1)	
*Marital Status (n = 12,758)*				
Never (ref)	116 (0.9)	21 (18.1)	95 (81.9)	0.0101
Married with spouse present	10,367 (81.2)	1731 (16.7)	8636 (83.3)	
Married not living with spouse	478 (3.8)	70 (14.6)	408 (85.4)	
Separated/Divorced/Widowed	65 (0.5)	8 (12.3)	57 (87.7)	
Divorced	102 (0.8)	14 (13.7)	88 (86.3)	
Widowed	1630 (12.8)	328 (20.1)	1302 (79.9)	
*Hypertension (n = 12,701)*				
No	9228 (72.7)	1482 (16.1)	7746 (60.9)	< 0.0001
Yes	3473 (27.3)	674 (19.4)	2799 (80.6)	
*Arthritis (n = 12,731)*				
No	8220 (64.6)	1172 (14.3)	7048 (85.7)	< 0.0001
Yes	4511 (35.4)	994 (22.0)	3517 (78.0)	
*Stroke (n = 12,725)*				
No	12,397 (97.4)	2077 (16.8)	10,320 (83.2)	< 0.0001
Yes	328 (2.6)	90 (27.4)	238 (72.6)	
*Diabetes (n = 12,641)*				
No	11,826 (93.5)	1960 (16.6)	9866 (83.4)	0.0011
Yes	815 (6.5)	193 (23.7)	622 (76.3)	
*Mental Impairment (n = 12,746)*				
No	12,421 (97.4)	2063 (16.6)	10,358 (83.4)	< 0.0001
Yes	325 (2.6)	106 (32.6)	219 (67.4)	
*Vision Impairment (n = 12,755)*				
No	11,835 (92.8)	1927 (16.3)	9908 (83.7)	< 0.0001
Yes	920 (7.2)	244 (26.5)	676 (73.5)	
		MEAN (SE)		
BMI	12,759	23.3 (0.10)	23.3 (0.04)	0.6233
Nap sleep duration/mins	12,759	31.1(0.90)	32.8 (0.42)	0.7519

^a^ in hours at night in the past month

^b^ in days per week

*SE* standard error, *ref* reference category

A large portion of the participants aged between 50 and 64 years (65.4%). Of the 6533(51.2%) females, 19.4% had falls, which is higher than 14.6% for males. The alcohol use, 8666(68.0%) was more common compared to their exposure to smoking 5191 (40.7%) among this study population. Among the psychosocial factors, more than half of the participants reported depression, about 21% reported anxiety and almost 30% reported loneliness. Almost one quarter of the participants who were either depressed, anxious or lonely most of the time (5–7 days) per week reported falls. The comorbidities considered in the present study revealed that almost 27% reported being diagnosed of hypertension, 35% diagnosed of arthritis, 2.6% diagnosed of stroke and 6.5% diagnosed of diabetes. Those who reported having mental and vision impairments constituted 2.6% and 7.2%, respectively.

The results of the bivariate analysis are presented in Table 1. Findings revealed the risk of falls is significantly higher for those who had short sleep duration ≤5 h than those who had long sleep duration ≥6. In addition, middle-aged and older adults who had their sleep disturbed more often were more likely to report falls. However, duration of nap after lunch did not show significant association with falls. Females were more likely to experience fall than males. The linearity assumption for the continuous covariates (age and BMI) were assessed by comparing the AICs for model with those covariates modeled as continuous variables versus categorical variables. The model with a smaller AIC indicates a better fit to the data. Our results indicated the AIC for the model with age as a categorical variable is smaller than the AIC for the model with age as continuous variable. However, the AIC for the model with BMI as a continuous variable had a smaller AIC when compared with the AIC of BMI modeled as categorical variable, so we proceed the analysis modeling age as categorical variable and BMI as continuous variable for the purpose of parsimony. As age increases, the risk of falls increases; whereas BMI is not significantly associated with falls. Exposure to alcohol did not show significant difference from those not exposed to these factors in relation to falls in the bivariate analysis. In contrast, there was a significant difference between those exposed to smoking and those not exposed in relation to falls. Psychosocial factors including depression, anxiety and number of days of loneliness were significantly associated with falls. The marital status of participants did show significant difference in falls in the bivariate analysis. All comorbidities were found to be significantly associated with falls in our study population.

In the final multivariable model (see Table 2), after adjusting for various psychosocial, lifestyle, socio-demographical characteristics and comorbidities, those participants who had sleep duration of ≤5 were more likely to report falls than those who slept 8 h (≤ 5 h: AOR = 1.34, 95% Cl 1.10–1.62). Those who had their sleep disturbed even as few as a day or two per week were more likely to report falls (AOR = 1.39, 95% Cl 1.09–1.77) and the odds of falls were also higher for those who had sleep mostly or occasionally disturbed (5–7 days per week: AOR = 1.40, 95% CI: 1.16–1.70; 3–4 days per week: AOR = 1.24: 1.02–1.50) than those who had no disturbed sleep. A dose response relationship was found between age and risk of falls. Thus, as age increases, the risk of falls also increases (age 65–74: AOR = 1.49, 95% CI 1.26–1.75; age 75+ years: AOR = 1.67, 95% CI 1.37–2.03). Males were less likely to report falls as compare to females (AOR = 0.71, 95% CI 0.61–0.82). Those who were exposed to alcohol were more likely to report fall than those with no exposure to alcohol (AOR = 1.53, 95% Cl 1.29–1.81). Participants who reported mostly depressed (5–7 days per week) and occasionally depressed (3–4 days per week) had a significantly higher risk of falls than those who had less than one-day depression (mostly depressed: AOR = 1.82, 95% CI 1.52–2.18; occasionally depressed: AOR = 1.41, 95% Cl 1.20–1.67). The participants who were diagnosed with hypertension (AOR = 1.23, 95% CI 1.09–1.50), arthritis (AOR = 1.57, 95% CI 1.36–1.82), and stroke (AOR = 1.64, 95% CI 1.12–2.41) had higher odds of reporting falls. Similarly, participants had elevated risk of falls if they had mental impairment (AOR = 1.95, 95% CI 1.44–2.65) and vision impairment (AOR = 1.48, 95% CI 1.21–1.82).

**Table 2:** Multivariable analysis assessing the associations of sleep duration and sleep disturbance with falls (*n* = 10,368)

Variables	Adjusted Odds Ratios (AOR)	95% Cl	*p*-value
*Sleep duration* ^*a*^ *(in hours)*			
≤ 5	1.34	(1.10–1.62)	0.0037
6–7	1.17	(0.98–1.40)	0.0924
8 (ref)			
9–10	1.38	(0.86–2.22)	0.1806
≥ 11	1.43	(0.74–2.74)	0.2867
*Sleep disturbance* ^*b*^			
None (<1 day) (ref)			
Little (1–2 days)	1.39	(1.09–1.77)	0.0091
Occasionally (3–4 days)	1.24	(1.02–1.50)	0.0293
Most (5–7 days)	1.40	(1.16–1.70)	0.0006
*Age in years*			
50–64 (ref)			
65–74	1.49	(1.26–1.75)	< 0.0001
75+	1.67	(1.37–2.03)	< 0.0001
*Gender*			
Female (ref)			
Male	0.71	(0.61–0.82)	< 0.0001
*Drinking Behaviour*			
No intake of alcohol (ref)			
Exposed to alcohol intake	1.53	(1.29–1.81)	< 0.0001
*Depression* ^*b*^			
None (< 1 day) (ref)			
Little (1–2 days)	1.14	(0.94–1.39)	0.1787
Occasionally (3–4 days)	1.41	(1.20–1.67)	< 0.0001
Most (5–7 days)	1.82	(1.52–2.18)	< 0.0001
*Hypertension*			
No (ref)			
Yes	1.23	(1.09–1.50)	0.0028
*Arthritis*			
No (ref)			
Yes	1.57	(1.36–1.82)	< 0.0001
*Stroke*			
No (ref)			
Yes	1.64	(1.12–2.41)	0.0115
*Mental Impairment*			
No (ref)			
Yes	1.95	(1.44–2.65)	< 0.0001
*Vision Impairment*			
No (ref)			
Yes	1.48	(1.21–1.82)	0.0002

^a^in hours at night in the past month

^b^in days per week

**Fig. 1:**
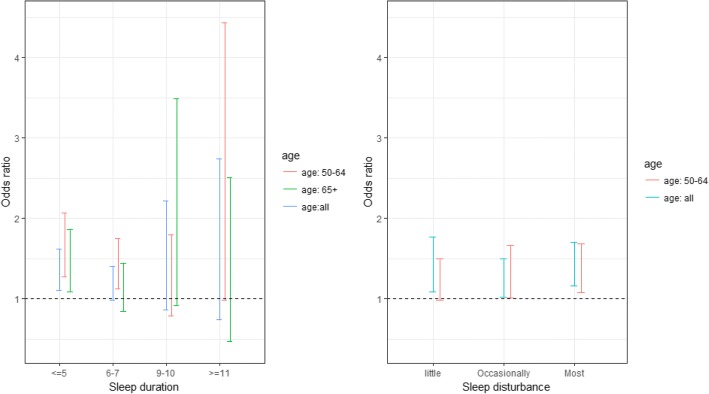
Odds ratio plots for falls in association with sleep duration and sleep disturbance based on the overall multivariate analysis and the stratified multivariate analyses by age group (age: 50–64 vs. age: 65+)

Tables 3 and 4 summarize the multivariate results of the association between sleep duration and sleep disturbance with falls by age groups (50–64 vs. 65+). After adjustment for psychosocial, lifestyle, socio-demographical characteristics and comorbidities, short sleep (duration of ≤5 h) among both aged 50–64 (AOR = 1.62; 95%CI 1.27–2.07) and aged 65+ (AOR = 1.42; 95%CI 1.09–1.86), were more likely to report falls than those who slept 8 h. In addition, we observed that participants aged 50–64 who had their sleep disturbed for 3–4 days (AOR = 1.30; 95%CI 1.01–1.67) and 5–7 days (AOR = 1.35; 95%CI 1.08–1.68) were more likely to fall. In contrast, sleep disturbance was not associated with falls among participants aged 65+. Figure 1 presents the odds ratio plots for falls in association with sleep duration and sleep disturbance based on the overall multivariate analysis and the stratified multivariate analyses by age groups (50–64 vs. 65+) for visual comparison.

**Table 3:** Multivariable analysis assessing the associations of sleep duration and sleep disturbance with falls (*n* = 8067) based on sample within the aged 50–64

Variables	Adjusted Odds Ratios (AOR)	95% Cl	*p*-value
*Sleep duration* ^*a*^ *(in hours)*			
≤ 5	1.62	(1.27–2.07)	0.0001
6–7	1.40	(1.12–1.75)	0.0030
8 (ref)			
9–10	1.20	(0.79–1.80)	0.3947
≥ 11	2.08	(0.98–4.43)	0.0576
*Sleep disturbance* ^*b*^			
None (<1 day) (ref)			
Little (1–2 days)	1.21	(0.98–1.50)	0.0827
Occasionally (3–4 days)	1.30	(1.01–1.67)	0.0436
Most (5–7 days)	1.35	(1.08–1.68)	0.0075
*Gender*			
Female (ref)			
Male	0.70	(0.57–0.87)	0.0009
*Depression* ^*b*^			
None (< 1 day) (ref)			
Little (1–2 days)	1.16	(0.96–1.42)	0.1340
Occasionally (3–4 days)	1.46	(1.18–1.81)	0.0006
Most (5–7 days)	2.13	(1.71–2.65)	< 0.0001
*Arthritis*			
No (ref)			
Yes	1.40	(1.21–1.63)	< 0.0001
*Stroke*			
No (ref)			
Yes	2.30	(1.50–3.52)	0.0001
*Mental Impairment*			
No (ref)			
Yes	1.74	(1.20–2.53)	0.0035
*Vision Impairment*			
No (ref)			
Yes	1.43	(1.09–1.89)	0.0115
*Smoking*			
Never			
Current	1.15	(0.92–1.43)	0.2183
Past	1.47	(1.09–1.98)	0.0122
*Marital Status*			
Never (ref)			
Married with spouse present	0.78	(0.38–1.59)	0.4863
Married not living with spouse	0.50	(0.22–1.10)	0.0861
Separated	0.22	(0.04–1.14)	0.0705
Divorced	0.23	(0.07–0.75)	0.0151
Widowed	0.65	(0.30–1.40)	0.2695

^a^ in hours at night in the past month

^b^ in days per week

**Table 4:** Multivariable analysis assessing the associations of sleep duration and sleep disturbance with falls (*n* = 4180) based sample with the aged 65 +

Variables	Adjusted Odds Ratios (AOR)	95% Cl	*p*-value
*Sleep duration* ^*a*^ *(in hours)*			
≤ 5	1.42	(1.09–1.86)	0.0107
6–7	1.10	(0.84–1.44)	0.4950
8 (ref)			
9–10	1.80	(0.92–3.49)	0.0853
≥ 11	1.09	(0.47–2.51)	0.8479
*Depression* ^*b*^			
None (< 1 day) (ref)			
Little (1–2 days)	1.22	(0.87–1.71)	0.2611
Occasionally (3–4 days)	1.50	(1.17–1.92)	0.0012
Most (5–7 days	1.51	(1.14–1.99)	0.0038
*Arthritis*			
No (ref)			
Yes	1.85	(1.46–2.34)	< 0.0001
*Mental Impairment*			
No (ref)			
Yes	2.79	(1.70–4.57)	< 0.0001
*Vision Impairment*			
No (ref)			
Yes	1.48	(1.11–1.97)	0.0071
*Hypertension*			
No (ref)			
Yes	1.30	(1.03–1.63)	0.0279

^a^ in hours at night in the past month

^b^ in days per week

Depression, arthritis, mental impairment and vision impairment were found to be consistently associated with falls, regardless of participants aged 50–64 or 65+, whereas other factors showed varied results depending on age groups. For instance, male gender (AOR = 0.70; 95%CI 0.57–0.87), stroke (AOR = 2.30; 95%CI 1.50–3.52) and past exposure to smoke (AOR = 1.47; 95%CI 1.09–1.98) were significantly associated with falls among participants aged 50–64; among participants aged 65+, hypertension showed a significant association with falls (AOR = 1.30; 95%CI 1.03–1.63).

## Discussion

Falls are increasingly recognised as a leading cause of disability and mortality worldwide. There has been limited research focusing on both sleep duration and disturbance as the primary risk factors for falls [51]. To the best our knowledge, few studies have been conducted examining the Chinese middle-aged and older adults. The primary finding in our study revealed that both sleep duration and sleep disturbance are strongly associated with falls after adjusting for various confounders**.** Further, our stratified analysis revealed that the effects of sleep duration and sleep disturbance on falls depend on age groups.

For sleep duration, our study found that sleep duration ≤5 h is significantly associated with increased risk of falls compared with sleep duration 6–7 h, which is consistent with literature [23, 24]. However, in the current study, long duration of sleep (> 8 h) was found to be not significantly related to falls. The latter finding has consistently been observed in previous studies [22, 24], whereas the other studies found long sleep duration (> 8 h) to be associated with increased risk of falls in older adults [25, 52]. In an attempt to verify the validity and reliability of the sleep duration variable in the present study data (CHARLS) in comparison with some other national data, such as Chinese Longitudinal Healthy Longevity Survey (CLHLS) [53], we made a comparison of the frequency distributions of the sleep duration variable between the CHARLS and CLHLS. In CHARLS, sleep duration variables considered nighttime sleep duration and nap sleep after lunch as separate variables; whereas CLHLS measured everyday sleep duration including napping [53]. Hence, sleep duration reported in CLHLS tends to be longer than those reported in CHARLS; as a result, less percentage of participants is expected to belong to the lower duration categories, but higher percentage of participants belonging to the higher duration categories in CLHLS as compared to CHALRS.

CLHLS focused on older adults of age ≥ 65, so to make the sleep duration variables between the CHARLS and CLHLS comparable, we considered participants aged ≥65 in CHARLS data and derived total sleep duration variable by combining nighttime sleep and nap sleep. The frequency distributions of sleep duration (Table 5) revealed CHARLS reported more participants (24.6%) who had sleep ≤5 as compared with CLHLS (13.1%), whereas CLHLS reported more participants (24.7%) had sleep duration ≥9 [53] compared with CHARLS (18.2%). The frequency of the rest of the categories were roughly comparable. The differences in the frequency distributions of the sleep duration variable between CHARLS and CLHLS may be due to the differences in the sampling provinces. CHARLS sampled 28 provinces and CLHLS sampled 22 provinces from China. A recent published meta-analysis on Chinese older adults revealed that five studied which reported sleep duration of less than 5 h (< 5 h) had a proportion of 18.8% whereas 15 studies that reported sleep duration less than 6 (< 6 h) had a proportion of 26.7% [21]. The differences observed could be partially attributed to the fact that the CHARLS included those who reported sleep duration of 5 h, which led to the higher proportion compared to studies that reported < 5 h and lower proportion compared to the studies that reported < 6 h. The studies that reported < 6 h might have included those who reported sleep duration more than 5 h but less than 6 h. Other differences could be due to the sample sized used, sampling methods used and where the studies were conducted (urban/rural/mixed). The sleep duration distribution in the CHARLS data therefore may not be the entirely representative of the sleep duration among middle-aged and older adults in China, so further studies are warranted in any attempts to generalize these results to other populations. Nevertheless, these findings contribute to the ongoing knowledge on how the sleep duration is associated with falls among middle-aged and older adults in China.

**Table 5:** Frequency distributions of sleep duration (in hours) in CHARLS vs. CLHLS for older adults of age 65 and above

CHARLS		CLHLS	
Sleep Duration in Hours	(night + nap after lunch)	Sleep Duration in Hours	(night + nap at any time)
≤ 5	24.6%	≤ 5	13.1%
6–7	32.5%	6	16.2%
		7	18.0%
8	24.7%	8	28.0%
9–10	9.1%	9	9.2%
		≥10	15.5%
≥ 11	9.1%		

Despite the potential population difference, sleep disturbances and other sleep-related comorbidities have been proposed as a plausible reason that may cause study findings to differ [25]. In the combined sample of the present study, sleep disturbance appeared to influence fall incidence, however, after splitting the study data into two samples, this association did not persist among participants aged 65+. The lack of association between sleep disturbance and falls among those aged 65+ could be partly due to sample size limitation, as larger samples would be needed to discover quite small differences [54]. The present study does not rule out the possibility of the association between sleep duration, sleep disturbance and fall being a bidirectional. A recent review on prospective studies ascertaining broadly risk factors for sleep disturbance did not identify fall as a potential risk factor for sleep disturbance [55]. However, sleep disturbance was rather found to be a risk factor of fall in other reviews investigating fall-related risk factors [56, 57]. The later finding suggests that sleep problems are more likely to precede incidence of fall. Napping behaviours may also contribute independently or influence risk of falls [50]. Existing literature therefore urged future studies to consider nap sleeping as part of sleep behaviours [58]. While existing literature found napping to be related to falls in elderly [50], this study found no significant association between napping after lunch and falls.

Our study also showed that falls are related with other factors including psychosocial, lifestyle, socio-demographic characteristics and comorbidity. These factors can either relate to falls independently or act in conjunction with other potential risk factors to impact on fall rates in middle-aged and older adults. For example, depressive symptoms were reported to be the strongest predictor of sleep disturbance [59] and sleep duration was shown to be linearly associated with prevalent and persistent psychological distress [60]. Other studies have also found increased risk of falls in elderly with depressive symptoms [61, 62]. Therefore, existing research evidence demonstrates the importance of controlling for such factors in any sleep-related research. The association between the middle-aged and older adults being depressed for 3 days or more and falls in the current study was consistent with published studies results [63, 64]. Although anxiety is less of a concern in older adult compared to younger people [65], a bivariate association between anxiety and falls was found in the present study. In addition, loneliness [66] has also been reported to be associated with falls in elderly subjects. Despite a crude association found between loneliness and falls in middle-aged and older adults in the present study, after adjusting for other variables, this association did not persist. However, the present study does not rule out the possibility of loneliness being associated with falls in middle-aged and older adults as biological mechanism including motor function decline [67] could play a role and may affect mobility [68]. For instance, Buchman et al. found “feeling alone and being alone” to be associated with “more rapid motor decline” in elderly subjects [67]. In addition, synergistic effect of loneliness and depression may contribute to more fall incidence among older adults, as both depression [61] and loneliness [66] have been found to be associated with falls in elderly population.

Lifestyle factors were also accounted for in the current study. Findings revealed that alcohol intake was significantly associated with falls in our study population. This also confirms findings by earlier study that participants without 12 months previous history of exposure to alcohol, after being exposed to small amount of alcohol were more likely to have sleep-related issues including “higher levels of sleep disturbances” [35]; which probably contributed to fall rate in this study population, as 68% of the population under study were exposed to alcohol. Although alcohol may independently relate to fall, there is evidence that alcohol use with other medications like benzodiazepines can rather “heighten the sedative effect of the medication” and hence increased fall rate [69]. Increased in fall rate has consistently been associated with age advancement [70]. The present study results on age and risk of falls was not an exception as a dose-response relationship was demonstrated between age and risk of falls. Similarly, Pearson et al. also found that as age increased both “risk and perceived risk of fall” also increased [71]. Only crude association was found between smoking and falls in the current study. Being female was found to be significantly related to increased risk of falls in older adults [24, 72].

In addition to life style and psychological factors influence on sleep related problems, other chronic conditions including arthritis, diabetes and stroke have been found to be associated with severe sleep problems in individuals 50 years and older [73]. These factors may contribute to sleep problem to increase fall rates in older adults [74, 75]. The present study accounted for several of these variables and found increased risk of fall in middle-aged and older adults with hypertension, stroke, arthritis, vision and mental impairments. The current study findings are consistent with earlier published results. James et al. found in increased risk of fall among older persons with hypertension [76]. Despite a crude association found between diabetes and fall in the current study, falls related to diabetes cannot be deemed as a mere chance. Strong association was found between diabetes and fall among elderly subjects in earlier published studies [76–78], Proposed mechanisms which diabetes mellitus could affect falls include “peripheral neuropathy, impaired vision due to diabetic retinopathy or cataracts and diabetic foot ulcers” [79]. Masil et al. also found increased risk of fall in elderly subjects with visual impairment [44], which highlights the importance of the present study accounting for vision impairment in the primary relationship between sleep related problems and fall in middle-aged and old adults. The present study also adopted the contextual model of elderly fall risk factors [80]. This model heighted the need for assessing the impact of mental health/cognitive in fall-related research. Hence this study assessed the impact of mental impairment including mental disorders and found that participants with mental impairment were at increased risk of fall. Mental impairment have consistently been found to be related with falls in older adults [45]. The literature also suggested that stroke is a major risk factor for falls. A recent prospective controlled study revealed greater risk of fall in an individual following a stroke when compared to those without stroke [81]. Likewise, increased risk of falling was also shown to be associated with arthritis among the elderly [82]. As such, for stroke survivors and middle-aged and older adults with arthritis, to reduce the risk of falling, interventions such as exercising or receiving physical therapy to improve walking speed, balance, and lower body strength have been recommended.

This study adds to existing evidence on fall by investigating the association of sleep duration and disturbances with fall using a robust procedure adjusting for complex survey design in a large sample [50]. The present study used a sample of the population-based nationwide data [46]. Although sleep variables including sleep duration and disturbance have been encouraged to be considered in sleep-related research as they may affect study results [25], a single study accounting for both variables in relation to fall in middle-aged and older adults is uncommon. Therefore, a notable strength of the present study is that it simultaneously investigates the association of both sleep duration and sleep disturbance with fall.

This study has a number of limitations. The use of cross-sectional design did not allow for causal inference to be drawn on the association between sleep duration and disturbances and fall. Besides, due to the time varying nature of both sleep parameters and fall, the study design used did not permit the ascertainment of which factor occurred first [83], however, paved the way for hypothesis generation which could be tested with rigorous study design. Also, this study assessed sleep disturbance on overall number of days experienced per week and did not allow for factors that led to the disturbance in sleep to be investigated independently. Although this study adjusted for several potential confounding variables, other factors including foot problems [84] and polypharmacy [15], which were reported to be related to fall were not accounted for in the current study, since no such data was collected. In addition, information collected from participants was based on self-report, so this study cannot rule out the possibility of recall bias. Despite the sleep questions were based on Pittsburgh Sleep Quality Index [39] and other questions derived from symptoms of sleep problems highlighted in literature [26, 42] and pilot tested, not all sleep questions stipulated in standardized tools such as Epworth Sleepiness scale [85] or Pittsburgh Sleep Quality Index [39] were considered in the CHARLS questionnaire.

## Conclusions

Results of the present study showed that after adjusting for the psychosocial, lifestyle and socio-demographical factors, sleep duration and disturbances were strongly and significantly associated with falls in Chinese middle-aged and older adults. Such significant associations underscore the need for evidence-based prevention and interventions for falls targeting sleep duration and disturbance among the Chinese middle-aged and older adults. Future prospective longitudinal study is warranted to investigate the causal relationship between sleep duration, sleep disturbances and falls.

## Abbreviations

AIC: Akaike Information Criterion
AOR: Adjusted Odds Ratios
BMI: Body Mass Index
CHARLS: China Health and Retirement Longitudinal Study
CI: Confidence Intervals
IRB: Institutional Review Board
PSQI: Pittsburgh Sleep Quality Index
WHO: World Health Organization
